# Aspirin inhibits the production of proangiogenic 15(*S*)-HETE by platelet cyclooxygenase-1

**DOI:** 10.1096/fj.201600530R

**Published:** 2016-09-15

**Authors:** Francesca Rauzi, Nicholas S. Kirkby, Matthew L. Edin, James Whiteford, Darryl C. Zeldin, Jane A. Mitchell, Timothy D. Warner

**Affiliations:** * The William Harvey Research Institute, Barts and the London School of Medicine and Dentistry, Queen Mary University of London, London, United Kingdom;; † National Heart and Lung Institute, Imperial College London, London, United Kingdom; and; ‡ National Institutes of Health, National Institute of Environmental Health Sciences, Research Triangle Park, North Carolina, USA

**Keywords:** angiogenesis, eicosanoids, endothelium, prostaglandins

## Abstract

Regular consumption of low-dose aspirin reduces the occurrence of colorectal, esophageal, stomach, and gastrointestinal cancers. The underlying mechanism is unknown but may be linked to inhibition of angiogenesis. Because the effective doses of aspirin are consistent with the inhibition of cyclooxygenase-1 in platelets, we used liquid chromatography with tandem mass spectrometry analyses and immunoassays of human platelet releasates coupled with angiogenesis assays to search for the mediators of these effects. Blood or platelet-rich plasma from healthy volunteers stimulated with platelet activators produced a broad range of eicosanoids. Notably, preincubation of platelets with aspirin, but not with a P2Y_12_ receptor antagonist, caused a marked reduction in the production of 11-hydroxyeicosatetraenoic acid (HETE) and 15(*S*)-HETE, in addition to prostanoids such as thromboxane A_2_. Releasates from activated platelets caused cell migration and tube formation in cultured human endothelial cells and stimulated the sprouting of rat aortic rings in culture. These proangiogenic effects were absent when platelets were treated with aspirin but returned by coincubation with exogenous 15(*S*)-HETE. These results reveal 15(*S*)-HETE as a major platelet cyclooxygenase-1 product with strong proangiogenic effects. Thus, 15(*S*)-HETE represents a potential target for the development of novel antiangiogenic therapeutics, and blockade of its production may provide a mechanism for the anticancer effects of aspirin.—Rauzi, F., Kirkby, N. S., Edin, M. L., Whiteford, J. Zeldin, D. C., Mitchell, J. A., Warner, T. D. Aspirin inhibits the production of proangiogenic 15(*S*)-HETE by platelet cyclooxygenase-1.

Epidemiologic data have established that regular consumption of aspirin reduces the occurrence of some cancers, notably colorectal, esophageal, stomach, and gastrointestinal (1–4). The mechanism underlying this remarkably significant effect is unknown, although it may be in part linked to reductions in angiogenesis.

Aspirin acts by irreversibly inhibiting the cyclooxygenase (COX) enzyme, and this explains its anti-inflammatory, analgesic, antipyretic, and antiplatelet effects (5–7). These effects of aspirin are produced at notably different dosages; the anti-inflammatory effects are seen only at very high dosage (many grams), the analgesic effects require high to medium dosage (around 600 mg), the antipyretic effects are exerted at medium dosage (300–600 mg), and the antiplatelet effects are fully achieved with a low dosage (75–100 mg) (7). Notably, the anticancer effects of aspirin are displayed at low dosages (4), consistent with those that inhibit platelets (8) and markedly below dosages required for analgesic or antiinflammatory effects.

Platelets are blood components central to hemostasis. Upon activation, platelets release large amounts of arachidonic acid (AA) from the membrane phospholipids largely through the activity of group IV A cytosolic phospholipase A_2_ (9, 10). The AA released is rapidly metabolized by multiple enzymatic pathways (9, 10). The best characterized of these pathways within platelets is the conversion of AA to thromboxane (TX)A_2_, which is dependent upon the actions of COX-1 and TX synthase. TXA_2_ is a potent prothrombotic hormone that drives platelet aggregation and thus the formation of blood thrombi. Regular consumption of low-dose aspirin irreversibly inhibits COX-1. This in turn blocks the formation of TXA_2_, which leads to reduced platelet activation and diminished thrombus formation. In contrast to the lack of insight regarding the ability of low-dose aspirin to reduce cancer, blockade of the production of TXA_2_ provides a clear mechanism to explain low-dose aspirin’s antithrombotic effectiveness (5–8).

Building upon the evidence that platelet COX-1 is the therapeutic target of low-dose aspirin, we characterized the eicosanoids produced by activated platelets and their sensitivities to clinically relevant antiplatelet levels of aspirin. The eicosanoids identified as major AA-derived platelet products were then investigated for their influence on cell targets within the vasculature (*i.e.,* platelets, leukocytes, and endothelial cells that could be relevant to the influences of aspirin upon cancer progression). From these studies we propose that 15(*S*)-hydroxyeicosatetraenoic acid (HETE) is a previously unidentified major COX-1 product of platelets whose inhibition could provide a mechanistic explanation for some of the anticancer effects of aspirin.

## MATERIALS AND METHODS

### Blood collection and ethics

Blood from healthy volunteers was collected by venepuncture into lepirudin (250 μg/ml). All experiments were subject to written informed consent and appropriate local ethical approval (healthy volunteer samples: St. Thomas’s Hospital Research Ethics Committee, reference 07/Q0702/24; patient samples: South East National Health Service Research Ethics Committee) and in accordance with Declaration of Helsinki principles. As reported previously, platelet counts were made to confirm that all samples were within the normal range (9, 11–13).

### Preparation of whole-blood and platelet-rich plasma samples for liquid chromatography with tandem mass spectrometry analysis

Blood was incubated with vehicle, aspirin (100 µM), the P2Y_12_ receptor antagonist prasugrel active metabolite (PAM) (3 µM; a gift from AstraZeneca), or aspirin + PAM. Part of the blood was centrifuged (175 *g*; 15 min) to obtain platelet-rich plasma (PRP). Platelets in both whole blood and PRP were then activated under static conditions (in initial experiments) or under stirring conditions in a light transmission aggregometer by the addition of collagen (30 µg/ml) (Takeda Pharmaceuticals, Deerfield, IL, USA), TRAP-6 (30 µM) (Sigma-Aldrich, St. Louis, MO, USA), or A23187 (50 µM) and incubation for 5 min. Plasma was then quickly separated from the samples by centrifugation (2000 *g*, 5 min, 4°C) and stored at −80°C for eicosanomic analysis, as previously described (14, 15). In brief, HyperSep Retain PEP SPE cartridges (Thermo Fisher Scientific, Waltham, MA, USA) were preconditioned with a solution of 0.1% acetic acid/5% methanol and spiked with 30 ng each of internal standards. Plasma (0.25 ml) was diluted in 0.1% acetic acid/5% methanol containing 0.009 mM butylated hydroxytoluene and added to the column. Samples were then washed with two volumes of 0.1% acetic acid/5% methanol, eluted in 1 ml of ethyl acetate and 1 ml methanol, dried by vacuum centrifugation at 37°C, and reconstituted in 30% ethanol. AA-derived metabolites were then separated by reverse-phase HPLC on a 1 × 150 mm, 5 μm Luna C18 (2) column (Phenomenex, Torrance, CA, USA) and quantified using a MDS Sciex API 3000 triple quadrupole mass spectrometer (Applied Biosystems, Foster City, CA, USA) with negative-mode electrospray ionization and multiple reaction monitoring. Data were captured and analyzed using Analyst 1.5.1 software. Relative response ratios of each analyte were used to calculate concentrations, and extraction efficiency for each sample was calculated based on recovery of the internal standards.

### Enzymatic immune assay for 15(*S*)-HETE

Enzymatic immune assay (EIA) assay was performed in accordance with the kit insert (Cayman Chemical Co., Ann Arbor, MI, USA). In brief, PRP and whole blood samples were diluted 1:10 and assayed in parallel to known 15(*S*)-HETE standards, a maximum binding control, nonspecific binding control, and blank on a 96-well plate coated with goat anti-IgG antibodies.

### Light transmission aggregometry

As we have reported previously (16), PRP prepared from blood treated with lepirudin was pretreated with aspirin (100 µM) (Sigma-Aldrich), PAM (3 µM), or aspirin + PAM and then incubated under stirring (1200 rpm; 1 min) before being stimulated with collagen (3 µg/ml) with or without the addition of U46619 (2 µM), prostaglandin (PG)D_2_/PGE_2_ (1 µM), 11(*R*)-HETE, 15(*S*)-HETE, and/or 15(*R*)-HETE (all 1 µM) (Cayman Chemical Co.). Aggregation was followed over 7 min, and the area under the curve (AUC) of platelet aggregation responses was determined.

### 96-well plate aggregometry

To measure responses to a broad panel of platelet agonists across a range of concentrations, we performed additional studies using 96-well plate-based aggregometry, as we have described previously (11, 13). In brief, 100-μl aliquots of PRP preincubated with aspirin (100 µM), PAM (3 µM), aspirin + PAM, or vehicle were loaded into 96-well microtiter plates containing A23187 (Ca^2+^ ionophore, 1.25–40 μM) (Sigma-Aldrich), ADP (0.1–30 μM; Sigma-Aldrich), collagen (0.1–30 μg/ml), epinephrine (1 nM–100 μM) (Labmedics/Chronolog, Oxfordshire, United Kingdom), TRAP-6 amide (0.25–5 μM), or the TXA_2_-mimetic U46619 (0.1–4 μM) (Enzo Lifesciences, Exeter, United Kingdom) with or without 5-HETE, 11-HETE, 12-HETE, or 15-HETE (1 µM). Plates were shaken vigorously for 16 min at 37°C in a microtiter plate reader (Sunrise; Tecan, Männedorf, Switzerland), during which time absorbance (595 nm) was recorded every 15 s. Aggregation (in percent) was calculated using the initial absorbance of each well (0%), and the absorbance of platelet-poor plasma as 100% reference.

### Neutrophil chemotaxis

Polymorphonuclear leukocytes were isolated from blood of healthy volunteers by centrifugation (400 *g*, 30 min) using a double-density gradient medium (Hystopaque 1109–Hystopaque 1007) and suspended at 4 × 10^6^ cells/ml in RPMI 1640 medium with 0.1% bovine serum albumin. The effects on neutrophil chemotaxis of leukotriene (LT)B_4_ at 1–100 nM, 5-HETE, 11-HETE, 12(*S*)-HETE, and 15-HETE at 0.3 and 3 µM and vehicle were studied using 96-well chemotaxis plates with a 3 µM pore size upper filter (3.2-mm diameter sites, 30-μl 96-well plate, 3 μm) (ChemoTx; Neuro Probe, Gaithersburg, MD, USA) incubated for 1.5 h (37°C, 5% CO_2_). Each experimental condition was assessed in quadruplicate, with cell migration determined by the colorimetric/fluorescent indicator AlamarBlue (Thermo Fisher Scientific).

### Tube formation assay

Immortalized human microvascular endothelial cells (line HMEC-1; Centers for Disease Control and Prevention, Atlanta, GA, USA) were cultured in RPMI 1640 medium with 10% fetal bovine serum (FBS) onto 48-well plates coated with 100 µl/well basement membrane extract in the presence of vehicle or 15(*S*)-HETE. Tube formation was visualized by time-lapse microscopy (magnitude, ×4) over 16 h using an inverted microscope (IX81; Olympus, Tokyo, Japan) with a digital camera (Orca-ER; Hamamatsu, Hamamatsu City, Japan). Image acquisition was performed using Cell M software (Olympus). After the 16 h incubation, images were taken in phase (original magnification, ×10) and in fluorescence (original magnification, ×10) by labeling cells with calcein AM. The number of branching points was counted after 4, 8, and 16 h incubation using Image J software (National Institutes of Health, Bethesda, MD, USA). In experiments investigating the effects of platelet incubates on HMEC-1 cells, platelets in PRP were activated under stirring conditions in a light transmission aggregometer by the addition of collagen (30 µg/ml) (Takeda) or TRAP-6 (30 µM) (Sigma-Aldrich) and incubation for 5 min [as for preparation for liquid chromatography with tandem mass spectrometry (LC-MS/MS) analysis] before being added directly to the cells together with an equal volume of RPMI 1640 medium + 2% FBS. For experiments investigating the effects of platelet releasates, plasma was quickly separated from the samples by centrifugation (2000 *g*, 5 min, 4°C) before combination with RPMI 1640 medium + 2% FBS and addition to the HMEC-1 cells.

### Cell migration assay

Confluent HMEC-1 cells on 6-well plates were scraped in a straight line using a p200 tip to create a “scratch” and incubated (37°C; 5% CO_2_) in RPMI 1640 medium with 10% FBS in the presence of vehicle or 15(*S*)-HETE. HMEC-1 migration was then visualized by time-lapse microscopy (magnitude, ×10) over 20 h using an Olympus IX81 inverted microscope with a Hamamatsu Orca-ER digital camera, and image acquisition was performed using Cell M software (Olympus). The percentage of area covered by cells throughout the 20 h incubation was calculated. Platelet releasates were prepared as previously described.

### Rat aortic ring assay

Thoracic aortas from 180–200 g Wistar rats (Charles River Laboratories, Wilmington, MA, USA) were sliced into equal sections and incubated (37°C; 5% CO_2_) overnight in serum-free Opti-MEM (Invitrogen, Carlsbad, CA, USA) before being embedded in type I collagen (1 mg/ml) in E4 medium (Invitrogen). Rings were then incubated (37°C; 5% CO_2_) in Opti-MEM (Thermo Fisher Scientific) plus 1% FBS (PAA Laboratories/GE Healthcare, Little Chalfont, United Kingdom) in the presence of platelet releasates and/or VEGF (10 nM), 15(*S*)-HETE (1 µM), or vehicle. Emergent angiogenic sprouts from rat aortas were counted after 4 d in culture. All animal experiments were conducted in accordance with the British Home Office regulation (Scientific Procedures) Act. Images were captured using an Olympus IX81 inverted microscope with a Hamamatsu Orca-ER digital camera, and image acquisition was performed using Cell M software (Olympus). Platelet releasates were prepared as described above.

## RESULTS

### Eicosanoid formation in whole blood and PRP

**Figure 1.**
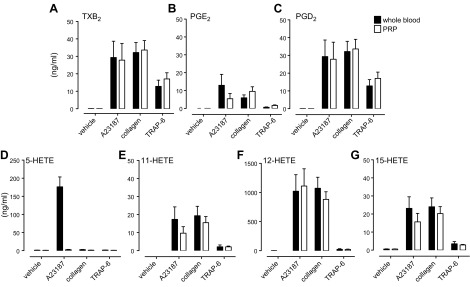
LC-MS/MS analysis of production of TXB_2_, PGE_2_, PGD_2_, 5-HETE, 11-HETE, 12-HETE, and 15-HETE in whole blood and PRP. Whole blood and PRP from healthy volunteers was incubated for 30 min at 37°C in the presence of Ca^2+^ ionophore (A23187; 50 µM), collagen (30 µg/ml), TRAP-6 (30 µM), or vehicle and then centrifuged (1300 *g*; −4°C) to produce plasma samples. Significant and similar increases in the levels of TXB_2_ (*A*), PGE_2_ (*B*), PGD_2_ (*C*), 5-HETE (*D*), 11-HETE (*E*), 12-HETE (*F*), and 15-HETE (*G*) were found in PRP and whole blood stimulated with A23187, collagen, or TRAP-6 compared with vehicle. The level of 5-HETE increased in whole blood only in response to A23187 but did not increase in PRP (*P* < 0.01; *n* = 5; 2-way ANOVA).

To test the source of eicosanoids produced in whole blood, incubations were made of blood or PRP from healthy volunteers together with A23187 (50 µM), collagen (30 µg/ml), TRAP-6 (30 µM), or PBS. Although a broad range of eicosanoids was detected (**Table 1**), those produced in the highest amounts to collagen and TRAP-6 were 12-HETE, TXA_2_, 15-HETE, and 11-HETE, with similar levels in PRP and whole blood (**Fig. 1**). 5-HETE was a very prominent product of whole blood stimulated with A23187 but was absent in PRP.

**TABLE 1.** LC-MS/MS analysis of production of eicosanoids in whole blood (pg/ml)

Eicosanoid	Vehicle	+ Collagen (30 µg/ml)	+TRAP-6 (30 µM)	+A23187 (50 µM)
12-HETE	8463 ± 4440	235,817 ± 55,654	43,267 ± 16,587	838,833 ± 77,940
TXB_2_	102 ± 32	17,618 ± 2704	24,072 ± 6528	38,433 ± 3835
15-HETE	749 ± 120	10,460 ± 1913	14,227 ± 4207	34,450 ± 2158
11-HETE	111 ± 18	4084 ± 711	6177 ± 2075	11,305 ± 1107
17,18-DHET	2633 ± 311	2825 ± 549	2844 ± 350	2995 ± 614
12,13-EpOME	2851 ± 494	2565 ± 356	2764 ± 466	2778 ± 475
13-HODE	1755 ± 237	1972 ± 251	1957 ± 261	3401 ± 496
PGF_2α_	1200 ± 92	1640 ± 143	1939 ± 294	1882 ± 42
5-HETE	697 ± 136	1270 ± 237	1212 ± 200	217,467 ± 34,158
PGE_2_	31 ± 8	1053 ± 182	2006 ± 875	4770 ± 1068
9-HODE	768 ± 106	1016 ± 100	1056 ± 148	1518 ± 93
19,20-DiHDPA	874 ± 147	941 ± 189	955 ± 182	907 ± 168
PGD_2_	17 ± 4	779 ± 195	1083 ± 438	2652 ± 543
5,6-EET	401 ± 68	647 ± 113	718 ± 77	2368 ± 120
14,15-DHET	461 ± 17	503 ± 42	497 ± 28	516 ± 41
19,20-EpDPE	420 ± 69	454 ± 51	465 ± 76	620 ± 126
9,12,13-THOME	1247 ± 768	340 ± 122	1210 ± 833	1180 ± 801
20-HETE	327 ± 89	336 ± 75	265 ± 97	389 ± 72
19-HETE	290 ± 14	315 ± 28	274 ± 18	312 ± 38
11,12-DHET	285 ± 15	302 ± 26	304 ± 22	313 ± 27
9,10-EpOME	311 ± 70	282 ± 26	311 ± 52	390 ± 45
8,9-DHET	147 ± 19	148 ± 23	152 ± 17	171 ± 27
14,15-EET	60 ± 4	113 ± 11	102 ± 7	402 ± 30
9,10,13-THOME	56 ± 15	108 ± 25	66 ± 23	162 ± 29
6ketoPGF_1α_	22 ± 5	96 ± 16	119 ± 34	248 ± 45
8,9-EET	72 ± 11	88 ± 5	97 ± 7	224 ± 11
5,6-DHET	51 ± 3	70 ± 19	62 ± 8	213 ± 68
8isoPGF_2α_	25 ± 1	63 ± 16	92 ± 25	110 ± 6
11,12-EET	19 ± 6	40 ± 2	48 ± 4	184 ± 9
17,18-EpETE	2 ± 1	1 ± 1	2 ± 1	12 ± 1

Whole blood was incubated under static conditions for 30 min at 37°C in the presence of vehicle control, collagen (30 µg/ml), TRAP-6 (30 µM), or Ca^2+^ ionophore (A23187; 50 µM) and then centrifuged (1300 *g*; −4°C) to produce plasma samples. Data are shown as means ± sem. Data are ordered by amounts of eicosanoids (pg/ml) produced in response to collagen (*n* = 4 for each).

### Eicosanoid formation in whole blood and PRP in the presence of antiplatelet treatment

**Figure 2.**
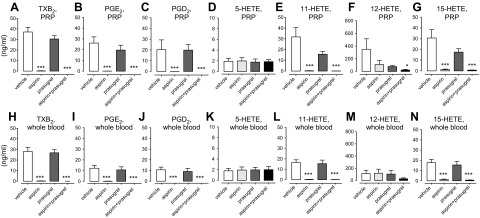
LC-MS/MS analysis of the production of TXA_2_, PGE_2_, PGD_2_, 5-HETE, 11-HETE, 12-HETE, and 15-HETE by PRP and whole blood in response to TRAP-6 in the presence of aspirin (100 µM), prasugrel (3 µM), or the combination of aspirin and prasugrel. Whole blood and PRP from healthy volunteers pretreated with aspirin (100 µM), prasugrel (3 µM), or the combination of aspirin and prasugrel was incubated for 30 min at 37°C in the presence of TRAP-6 (30 µM) or vehicle and then centrifuged (1300 *g*; −4°C) to produce plasma samples. Stimulation of PRP (*A*–*G*) and whole blood (*H*–*N*) with TRAP-6 significantly increased the levels of TXA_2_ (*A*, *H*), PGE_2_ (*B*, *I*), PGD_2_ (*C*, *J*), 5-HETE (*D*, *K*), 11-HETE (*E*, *L*), 12-HETE (*F*, *M*), and 15-HETE (*G*, *N*), which were fully inhibited by aspirin or the combination of aspirin and prasugrel. The levels of 5-HETE and 12-HETE were not affected by the presence of any treatment in either PRP or whole blood, except for the production of 12-HETE, which was significantly inhibited by aspirin and prasugrel in combination. Data are shown as means ± sem (ANOVA with Dunnett posttest; *n* = 4). *P < 0.05; ***P < 0.001.

Focusing on the major eicosanoid products identified above, aspirin alone or in combination with prasugrel blocked the production of TXA_2_, PGE_2_, and PGD_2_, along with 11-HETE and 15-HETE, in whole blood and in PRP. EIA indicated that the 15(*S*)-HETE enantiomer was a major product and that aspirin inhibited its formation (**Table 2**). The addition of 15(*R*)-HETE standards to the EIA verified that the assay was highly selective for 15(*S*)-HETE, providing further confirmation that 15(*S*)-HETE was produced by the activated platelets. The productions of 12-HETE in blood and PRP were not significantly affected by aspirin or PAM but were reduced by the combination of aspirin and PAM (**Fig. 2**). Levels of 5-HETE did not increase after stimulation of blood or PRP with collagen or TRAP-6 and were not affected by any of the treatments tested.

**TABLE 2.** Production of 15(*S*)-HETE (ng/ml) by platelets in response to collagen or TRAP-6 in the presence of vehicle, aspirin, PAM, or aspirin + PAM

Treatment	Collagen (30 μg/ml)		TRAP-6 (30 μM)	
	PRP	Whole blood	PRP	Whole blood
Control	22.6 ± 0.8	14.3 ± 2.2	10.4 ± 1.2	8.8 ± 0.7
+ Aspirin	7.7 ± 1.1*	6.2 ± 1.0*	3.8 ± 0.7*	3.5 ± 0.3*
+ PAM	16.2 ± 0.8*	14.4 ± 1.2	9.2 ± 1.7	7.8 ± 0.8
+ Aspirin + PAM	5.6 ± 0.7*	5.0 ± 0.6*	2.9 ± 0.5*	3.1 ± 0.2*

Data obtained by ANOVA with Dunnett posttest (*n* = 4 for all). Data are shown as means ± sem. *P < 0.05.

### Effect of platelet COX-1-derived prostanoids and 11(*R*)-, 15(*S*,*R*)-HETE on platelet aggregation

Having established 11-HETE and 15(*S*)-HETE as platelet COX-1 products, the effects of these on platelet aggregation were tested alongside the other COX-1 products at the proportions identified by LC-MS/MS analysis (*i.e.,* these experiments were aimed to clarify the net effect of the combination of COX-1-dependent products upon platelet aggregation). Aggregation induced by collagen (3 µg/ml) in the presence of vehicle (light transmission aggregometry; AUC, 503 ± 118) was greatly reduced by aspirin (AUC, 92 ± 18) and largely restored by the addition of U46619 (2 µM) as a surrogate for TXA_2_ (AUC, 352 ± 86). This effect was blunted by further addition of PGE_2_ (1 µM) and PGD_2_ (1 µM) (TXA_2_/PGE_2_/PGD_2_, 2:1:1; AUC, 176 ± 42) but was unaffected by further addition of the HETE enantiomers (1 µM) [TXA_2_/PGE_2_/PGD_2_/11(*R*)-HETE/15(*R*)-HETE/15(*S*)-HETE, 2:1:1:1:1:1; AUC, 141 ± 25] (n = 4 for all). Neither exogenous 11-HETE, 12(*S*)-HETE, 15(*S*)-HETE, 5-HETE (all 1 µM), nor 12-LOX inhibition with baicalein or nordihydroguaiaretic acid (both 10 µM) affected platelet aggregation to collagen or TRAP-6 (data not shown).

### Effect of HETE isoforms on neutrophil chemotaxis

**Figure 3.**
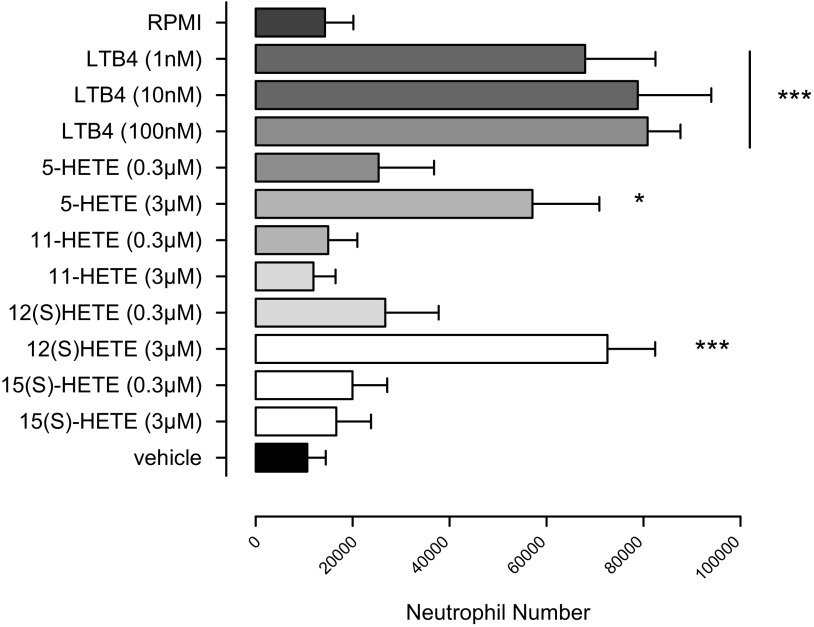
Effects of LTB_4_, 11-HETE, 15-HETE, 5-HETE, and the 12(*S*)-HETE stereoisomer on neutrophil chemotaxis. The chemotactic responses of isolated neutrophils obtained from human blood and stimulated with 11-HETE, 15-HETE, 5-HETE, 12(*S*)-HETE (0.3–3 µM), or LTB_4_ (1–100 nM, as control) were studied using a disposable Chemotaxis System. LTB_4_ (1, 10, or 100 nM) significantly induced neutrophil chemotaxis, as did 5-HETE (3 µM) and 12(*S*)-HETE (3 µM), whereas 11-HETE and 15-HETE were without effects. Data are shown as means ± sem (ANOVA with Dunnett posttest; *n* = 4). *P < 0.05; ***P < 0.01.

The effects on neutrophil chemotaxis of 11(*R*)-HETE, 15(*S*)-HETE, 5-HETE, and of the 12(*S*)-HETE stereoisomer (0.3–3 µM) were compared with those of LTB_4_, a potent neutrophil chemoattractant. LTB_4_ (1–100 nM) produced a strong chemoattractant effect on neutrophils, inducing migration of 80–90% of cells (**Fig. 3**). 5-HETE (3 µM) and 12(*S*)-HETE (3 µM) also exhibited strong chemotactic effects on 50–70% of neutrophils, but 11(*R*)-HETE and 15(*S*)-HETE were without effect.

### Effect of incubates of platelets activated by collagen or TRAP-6 in the presence or absence of aspirin on HMEC-1 tube formation

**Figure 4.**
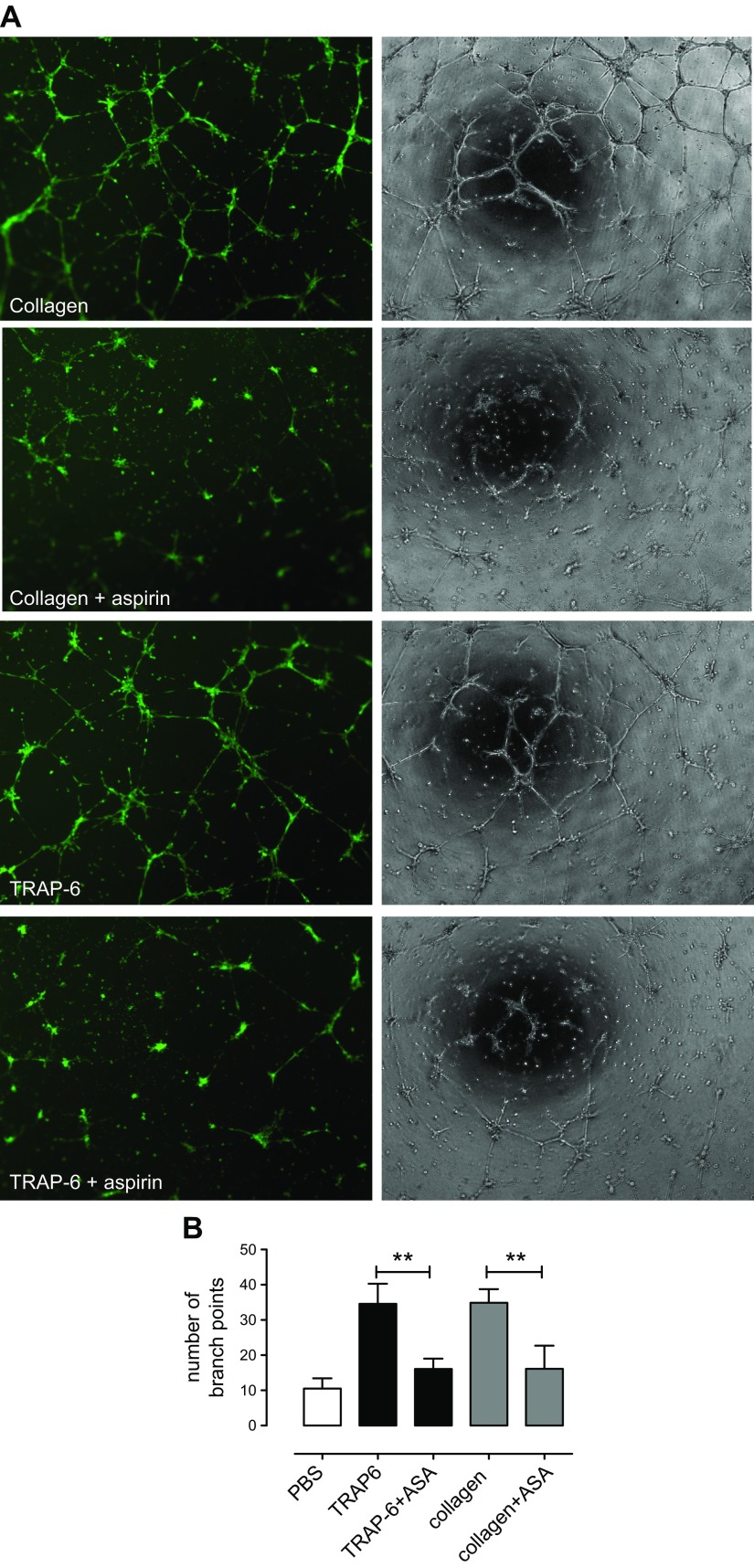
Effect of incubates of platelets activated by collagen or TRAP-6 in the presence or absence of aspirin on HMEC-1 tube formation. HMEC-1 cells were resuspended in platelet incubates diluted with RPMI 1640 medium + 2% FBS and (1:1) and seeded (40,000 cells/well) on a 48-well plate coated with growth factor-reduced basement matrix extract. Platelet incubates contained human PRP stimulated with collagen (30 µg/ml) or TRAP-6 (30 µM) in the presence or absence of aspirin (100 µM) which were added to the cell suspensions. *A*) Tube formation was visualized using time-lapse microscopy, where images were taken in phase every 30 min for 16 h (Cell M software). Images (original magnification, ×10) were taken after 16 h incubation (37°C; 5% CO_2_) in phase or after the addition of calcein AM. *B*) The number of branching points was quantitated after 16 h incubation using ImageJ software. Collagen-stimulated or TRAP-6-stimulated platelets significantly increased HMEC-1 tube formation. This effect was markedly reduced in response to aspirin-treated platelets. Data are shown as means ± sem (1-way ANOVA; *n* = 6). *P < 0.05.

Incubates of platelets stimulated with TRAP-6 (30 µM) or collagen (30 µg/ml) robustly promoted tube formation of HMEC-1 cells (**Fig. 4**), but this effect was absent when platelets were pretreated with aspirin (Fig. 4).

### Effect of 15(*S*)-HETE on HMEC-1 tube formation in the presence of releasates from aspirin- or vehicle-treated platelets stimulated with TRAP-6 or collagen

**Figure 5.**
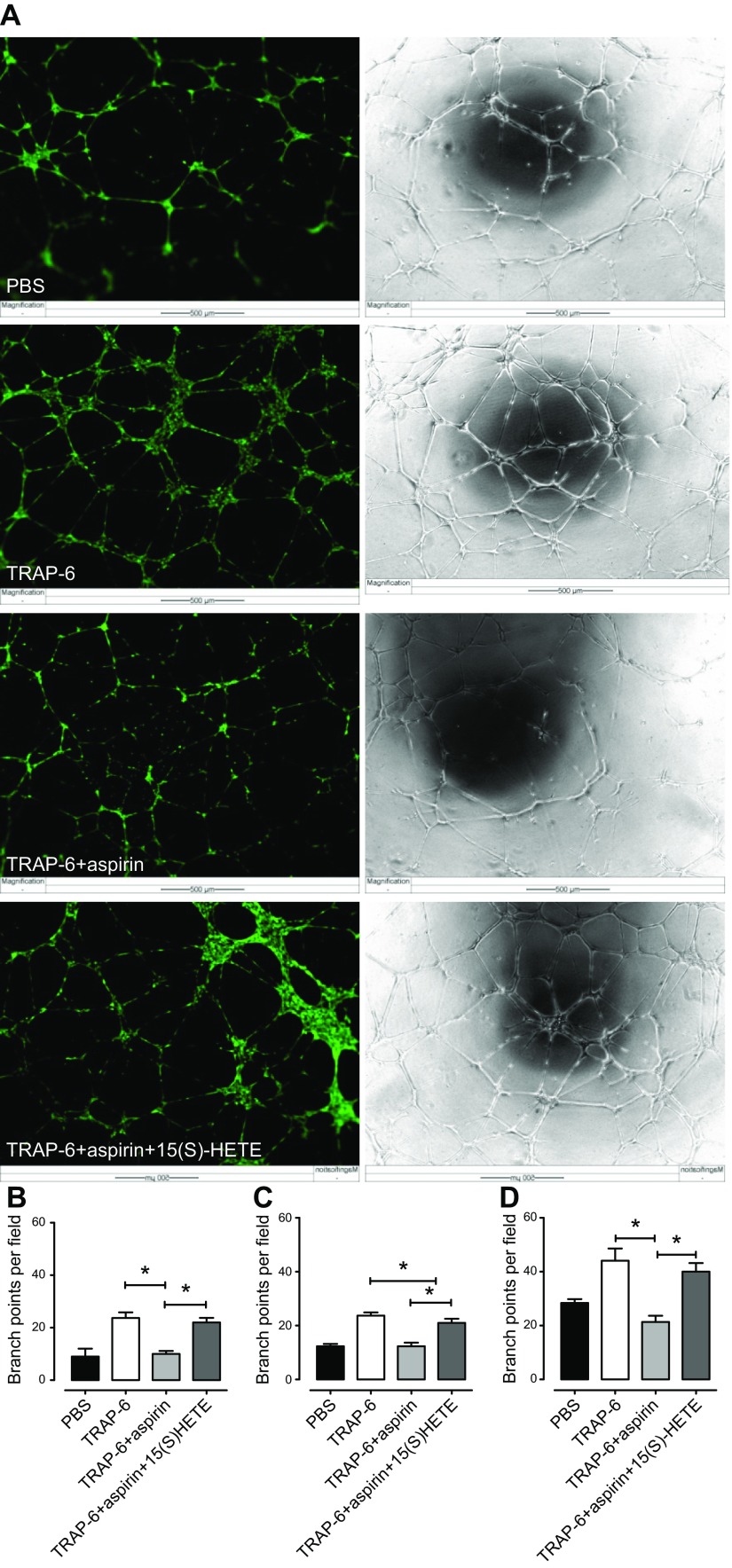
Effect of 15(*S*)-HETE on HMEC-1 tube formation in the presence of TRAP-6-stimulated platelet releasates. HMEC-1 cells were resuspended in platelet releasates diluted with RPMI 1640 medium + 2% FBS and (1:1) and seeded (40,000 cells/well) on a 48-well plate coated with growth factor-reduced basement matrix extract. Platelet releasates were obtained by centrifugation of human PRP stimulated with PBS or TRAP-6 (30 µM) in the presence or absence of aspirin (100 µM). The HMEC-1 cell suspensions were then treated with platelet releasates plus vehicle or 15(*S*)-HETE (1 µM). *A*) Tube formation was visualized using time-lapse microscopy, where images were taken in phase every 30 min for 16 h (Cell M software). Images (original magnification, ×10) were taken after 16 h incubation (37°C; 5% CO_2_) in phase or after the addition of calcein AM. *B*–*D*) The number of branching points was quantitated after 4 h (*B*), 8 h (*C*), and 16 h (*D*) incubation using ImageJ software. TRAP-6-stimulated platelet releasates significantly increased HMEC-1 tube formation. This effect was markedly reduced in response to releasates prepared from aspirin-treated platelets, but fully restored by addition of 15(*S*)-HETE (1 µM). Data shown as means ± sem (1-way ANOVA; *n* = 3). *P < 0.05.

Releasates from platelets stimulated with TRAP-6 (30 µM) or collagen (30 µg/ml) robustly promoted tube formation of HMEC-1 cells (**Fig. 5**). This effect was strongly impaired in releasates from aspirin-treated platelets but was fully restored by the addition of 15(*S*)-HETE (1 µM). 15(*S*)-HETE (1 µM) did not increase the proangiogenic effects of releasates from non-aspirin-treated platelets.

### Effect of 15(*S*)-HETE on HMEC-1 migration in the presence of releasates from aspirin- or vehicle-treated platelets stimulated with TRAP-6 or collagen

Releasates from platelets stimulated with TRAP-6 produced a small but significant increase in cell migration in the scratch assay (field cover: control, 55 ± 4%; +TRAP-6, 67 ± 4%; *P* < 0.05). This effect was absent in response to releasates from aspirin-treated platelets stimulated with TRAP-6 (48 ± 3%) but was restored by the addition of 15(*S*)-HETE (1 µM) (58 ± 4%; *P* < 0.05) (*n* = 4 for all).

### Effect of 15(*S*)-HETE on formation of sprouts from rat aortic rings in the presence of releasates from aspirin- or vehicle-treated platelets stimulated with TRAP-6 or collagen

**Figure 6.**
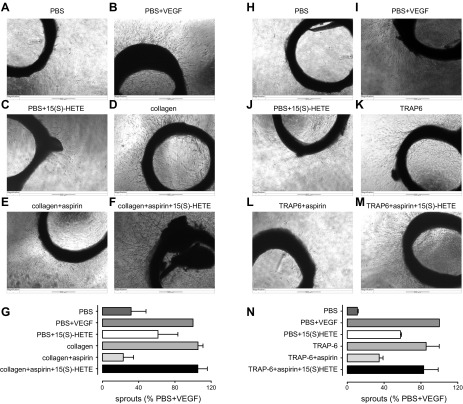
Effect of 15(*S*)-HETE on formation of angiogenic sprouts from rat aortic rings in the presence of collagen or TRAP-6-stimulated platelet releasates. Platelet releasates prepared as in Fig. 5 were diluted in Opti-MEM + 1% FBS (1:3) with the addition of supplemental collagen or TRAP-6 and/or aspirin to provide common incubation solutions for all rings. Rings incubated with PBS were treated without (*A*, *H*) or with (*B*, *I*) VEGF (10 ng/ml) or 15(*S*)-HETE (1 µM) (*C*, *J*), whereas rings incubated with releasates from platelets treated with collagen (*D*–*F*) or TRAP-6 (*K*–*M*) were treated without or with 15(*S*)-HETE (1 µM). Once additions had been made, aortic rings (5/condition) were incubated (37°C, 5% CO_2_) for 5 d. Images were taken in phase (original magnification, ×4). *G*, *N*) The number of sprouts was determined using Cell Ml software. *A*, *H*) PBS alone did not stimulate formation of sprouts. *B*, *C*, *I*, *J*) In contrast, the presence of VEGF significantly increased the number of sprouts (*B*, *I*), whereas 15(*S*)-HETE produced a moderate increase (*C*, *J*). *D*, *E*, *K*, *L*) Releasates from platelets stimulated with collagen (*D*) or TRAP-6 (*K*) significantly promoted the formation of sprouts, whereas releasates from platelets treated with aspirin had much weaker effects (*E*, *L*). *F*, *M*) The addition of 15(*S*)-HETE (1 µM) to aspirin-treated platelet releasates restored the formation of angiogenic sprouts. One-sample test used to compare differences to control response (PBS + VEGF; *n* = 3 for each). *P < 0.05.

Incubation of rat aortic rings with releasates from platelets stimulated with collagen or TRAP-6 led to robust increases in the number of sprouts comparable to those caused by VEGF, a well-characterized proangiogenic factor (**Fig. 6**). However, the number of sprouts was strongly reduced in aortic rings incubated with releasates from platelets that had been treated with aspirin. The loss of sprout-stimulating activity was restored by the addition of 15(*S*)-HETE (1 µM) to the aspirin-treated releasates.

## DISCUSSION

Although the use of low-dose aspirin for the secondary prevention of vascular thrombotic events has been established for many years (8), it is only recently that epidemiologic and observational analyses have revealed that long-term use of aspirin at low doses confers protection against particular types of cancers, notably colorectal, esophageal, stomach, and gastrointestinal (1–4). As outlined above, aspirin at low dose exerts its strongest effect on platelets, with higher doses being required for systemic analgesic and anti-inflammatory effects. Although the mechanism of action of aspirin in reducing cancer progression is not clear, the rational conclusion to be drawn from available epidemiologic and pharmacokinetic data is that much of this beneficial effect is due to inhibition of platelet COX-1. Therefore, we hypothesized that exposure of platelets to aspirin limited the production of procancerous and/or proangiogenic factors associated with COX-1. To test this concept, we conducted an unbiased LC-MS/MS analysis of the products of blood and platelet-rich plasma exposed to known platelet activators in control conditions in the presence of aspirin and in the presence of the platelet inhibitor PAM, which does not inhibit COX-1. We conducted incubations under stirring conditions because this better predicts platelet reactivity in the body than static conditions (17). As others and we have reported previously (9, 18, 19), activated platelets produce a broad range of AA-derived eicosanoids. Notably, in our studies we found 11-HETE and 15-HETE to be major products formed at similar levels to TXA_2_ through the action of group IV A cytosolic phospholipase A_2_ and COX-1. Despite not having been previously reported as platelet COX-1 products, this finding is consistent with an earlier report that isolated COX-1 enzyme can directly produce 11(*R*)-HETE, 15(*R*)-HETE, and 15(*S*)-HETE when AA is present within the active site of the enzyme in different catalytically competent arrangements (20). We hypothesize that this effect may be seen in strongly activated platelets where very high levels of AA can be released. Because our LC-MS/MS analyses could not discriminate 15(*R*)-HETE from 15(*S*)-HETE, we used a discriminatory immunoassay, which demonstrated 15(*S*)-HETE as a major product.

In our studies we also noted that in blood, but not PRP, the formation of 5-HETE increased robustly in response to Ca^2+^ ionophore (A23187) but not to the selective platelet activators collagen or TRAP-6. This finding is consistent with 5-HETE being a leukocyte product dependent upon the activity of the 5-LOX enzyme and provides a useful control for the selectivity and relevance of our assays. Notably, and consistent with the discriminatory abilities of our assays, the levels of 12-HETE in both blood and PRP were not affected by the presence of aspirin or prasugrel alone but were reduced when the drugs were used in combination. This result is consistent with dual antiplatelet therapy providing a general inhibition of platelet activation with the consequent reduction of AA release from the membrane phospholipids and thus decreasing the levels of 12-HETE. The lack of effect of aspirin against the production of 12-HETE provides further evidence for the drug acting selectively on COX-1 in our assays. Likewise, the lack of effect of PAM against the production of 11-HETE and 15-HETE supports the conclusion that these are COX-1-dependent products (*i.e.*, the inhibition of their production caused by aspirin is not explained by a reduction in platelet reactivity).

Having established that 11-HETE and 15(*S*)-HETE are major COX-1 and aspirin-sensitive products of platelets, we next sought to determine their functional roles. We reasoned that the prime cellular targets of these hormones were within the vasculature; thus, we focused on platelets, leukocytes, and vascular endothelial cells. We studied the effects of exogenous 11(*R*)-HETE, 15(*S*)-HETE, and 15(*R*)-HETE alongside the other COX-1 products we had identified on platelet aggregation *in vitro* using the proportions found by our LC-MS/MS analysis and concentrations previously reported as having relevant biologic activity (12, 13). Particularly, we tested the TXA_2_-mimetic (U46619), PGE_2_, and PGD_2_ together with 11(*R*)-HETE, 15(*S*)-HETE, and 15(*R*)-HETE on platelets treated with aspirin to block the effects of endogenous COX-1 products before stimulation with aspirin-sensitive concentrations of collagen. We found that U46619 restored aggregation, which was then partially inhibited by further addition of PGE_2_ and PGD_2_. This confirmed the already well-known strong proaggregatory effects of TXA_2_, and the inhibitory effects of PGD_2_ and high concentrations of PGE_2_, on platelet aggregation. Addition of 11(*R*)-HETE, 15(*R*)-HETE, and 15(*S*)-HETE in proportions matching those detected by LC-MS/MS did not alter these aggregation responses. We noted in these studies that the levels of TXB_2_ detected by LC-MS/MS was of the order of 10-fold lower than the concentrations of U46619 required to produce platelet activation (≈0.1 vs. 2 µM). This is consistent with previous studies and may be explained by the differences in sampling from the entire incubate volume compared with the amounts of mediators present in the small spaces between interacting platelets. Therefore, we focused upon the relative proportions of the mediators to be added to the platelets. Overall, these studies characterized the individual and net effects of platelet COX-1 products on platelet activity and demonstrated that these are not responses regulated by 11(*R*)-HETE, 15(*R*)-HETE, or 15(*S*)-HETE when considered in a proportionate manner to COX-1 products known to be active, particularly TXA_2_, PGD_2_, and PGE_2_.

Having established a lack of effect of 11(*R*)-HETE, 15(*R*)-HETE, and 15(*S*)-HETE on acute platelet responses, we next assessed their effects on inflammatory cells because HETEs are known to modulate neutrophil trafficking and recruitment (21). We found that although 5-HETE and 12(*S*)-HETE produced strong chemoattractant effects on human neutrophils, as did the potent neutrophil-derived chemoattractant LTB_4_, 11(*R*)-HETE, 15(*R*)-HETE, and 15(*S*)-HETE were without effect.

Based on the evidence that 15(*S*)-HETE promotes angiogenic responses in both human dermal microvascular endothelial cells and human umbilical vein endothelial cells by up-regulating VEGF through the PIK3-Akt and p38 MAPK signaling pathways, we next assessed whether this enantiomer could be involved in angiogenic responses using both *in vitro* and *ex vivo* models of angiogenesis (22–25). Etulain *et al*. (26) have previously reported an aspirin-sensitive angiogenic effect of platelet releasates upon HMEC-1 cells. Using the same cell line, we similarly found that incubates of activated platelets strongly promoted the formation of angiogenic tubules and that this was significantly reduced when platelets had been pretreated with aspirin. To explore these interactions further, we centrifuged the activated platelet preparations before addition to the HMEC-1 cells to focus particularly on the platelet releasates produced during platelet activation and not on other products or platelet components that could interact with the cells over the much longer periods of cell culture required to study indicators of angiogenesis. With regard to the platelet incubates, releasates obtained from agonist-stimulated platelets strongly promoted the formation of angiogenic tubules by HMEC-1 cells in an aspirin-sensitive manner, indicating that activated platelets acutely produce a proangiogenic factor through the activity of COX-1. We could then fully restore the response by the addition of exogenous 15(*S*)-HETE at the concentrations tested above, suggesting that 15(*S*)-HETE was the COX-1-dependent proangiogenic product. A similar outcome was seen in HMEC-1 migration. In direct accord with these results using cultured cells, our *ex vivo* studies clearly demonstrated that sprout formation from rat aortic rings was promoted by releasates from stimulated platelets but not by releasates prepared from platelets treated with aspirin. Addition of exogenous 15(*S*)-HETE rescued this process from aspirin inhibition.

In summary, our results suggest that 15(*S*)-HETE, formed as a direct COX-1 product, in combination with other proangiogenic factors released by activated platelets (*e.g.*, VEGF and PDGF), is central to regulating endothelial angiogenesis, particularly the formation of tubule structures that represent the core of new vessels. It has been established for many years that inhibition of platelet COX-1 and consequent reduction in TXA_2_ production explains the ability of low-dose aspirin to reduce thrombotic events. We suggest that the inhibition of 15(*S*)-HETE production by platelet COX-1 reducing angiogenesis could provide a similarly clear explanation for some of the cancer protective effects of low-dose aspirin.

## Abbreviations

AA: arachidonic acid
AUC: area under the curve
COX: cyclooxygenase
EIA: enzymatic immune assay
FBS: fetal bovine serum
HETE: hydroxyeicosatetraenoic acid
LC-MS/MS: liquid chromatography with tandem mass spectrometry
LT: leukotriene
PAM: prasugrel active metabolite
PG: prostaglandin
PRP: platelet-rich plasma
TX: thromboxane
